# Supplementary material for: Glycan-dependent cell adhesion mechanism of Tc toxins
Source: Nat Commun. 2020 Jun 1;11:2694. doi: 10.1038/s41467-020-16536-7 (PMC7264150; doi:10.1038/s41467-020-16536-7)
Supplement: Supplementary file 1 — Supplementary Information [file 41467_2020_16536_MOESM1_ESM.pdf]

Supplementary Information

Glycan-dependent cell adhesion mechanism of Tc toxins

Daniel Roderer et al.

Supplementary Figures

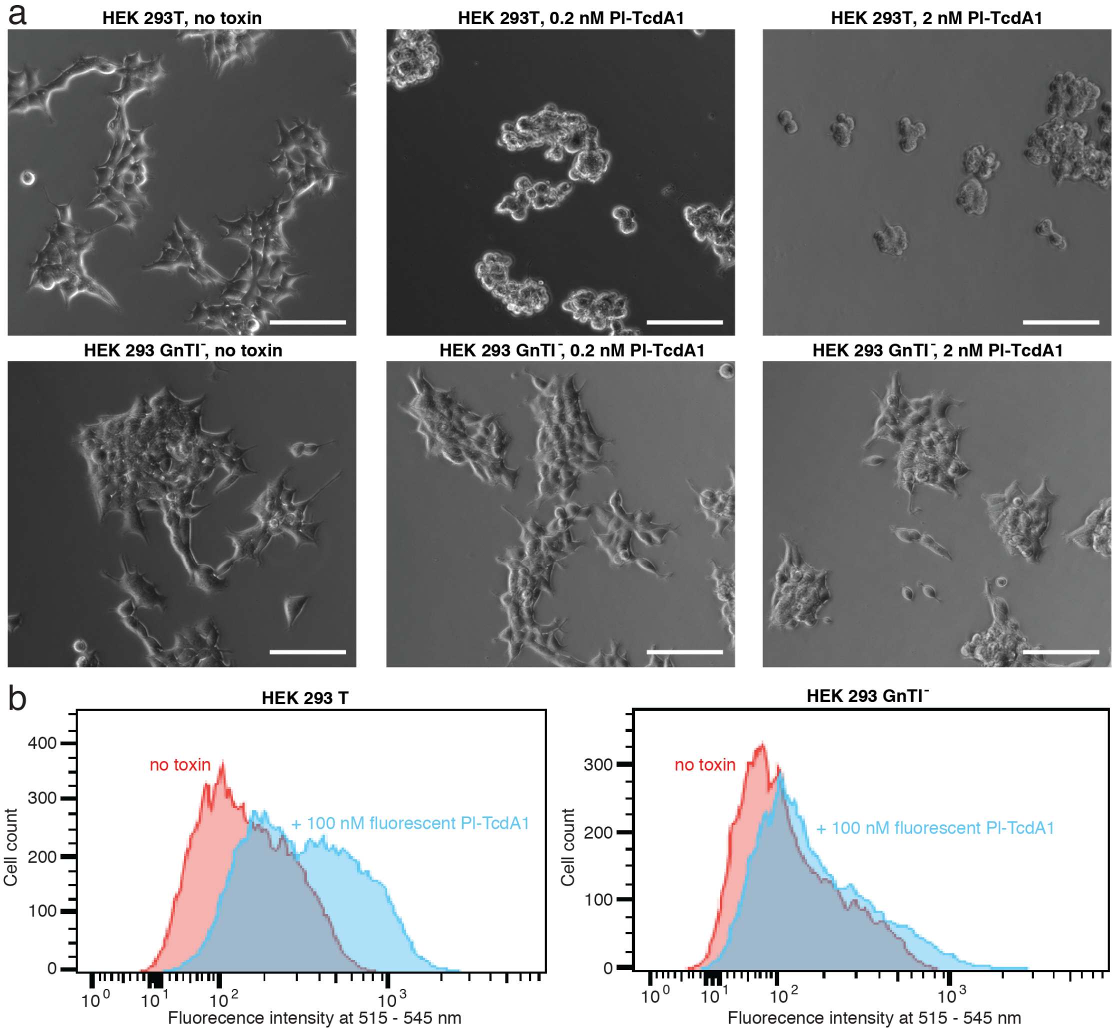

Supplementary Figure 1: Toxicity and binding of Tc to HEK 293 T and HEK 293 GnTI- cells. a: Intoxication of HEK 293T (top row) and HEK 293 GnTI- (bottom row) with Tc holotoxin (Pl-TcdA1 with Pl-TcdB2-TccC3). Images show cells 16 h after intoxication. Intoxicated cells round up and detach from the surface. Experiments were performed in triplicates with qualitatively identical results. Scale bars, 100 µm. b: Flow cytometry of HEK 293T (left) and HEK 293 GnTI- (right) exposed to AlexaFluor488 labelled Pl-TcdA1. Histograms of cells, both with (blue) and without (red) 100 nM Pl-TcdA1 are shown in comparison.

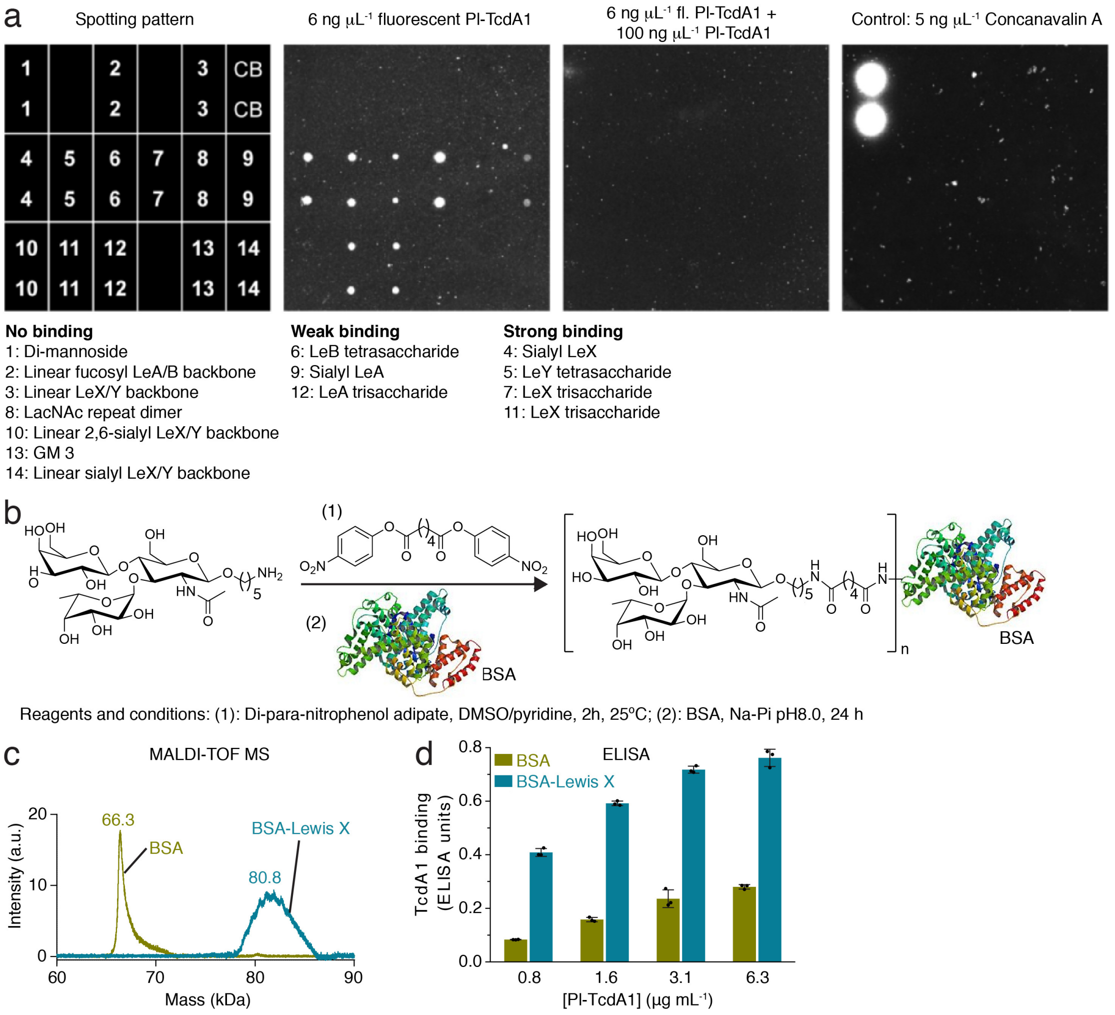

Supplementary Figure 2: Glycan microarray of Pl-TcdA1 and preparation of BSA-Lewis X. a: Spotting pattern of glycans on the chip surface in duplicates (left), fluorescence readout after incubation with 6 ng μL^{-1} fluorescently labeled Pl-TcdA1 alone (middle left) and together with a 17-fold excess of unlabeled Pl-TcdA1 (middle right), and fluorescence readout after incubation with concanavalin A (right). The glycans immobilized on the chip (structures are shown in Figure 2c) are shown below the chip scheme and are grouped according to their interaction with Pl-TcdA1. b: Reaction scheme showing the preparation of BSA-Lewis X, which was used for BLI and cryo-EM. BSA: bovine serum albumin. c: Mass spectrometry (MALDI-TOF MS) of the obtained BSA-Lewis X glycoconjugate in comparison to BSA. The average mass increase of 14.5 kDa shows the immobilization of ~20 Lewis X trisaccharides per BSA molecule. d: ELISA of Pl-TcdA1 (0.8 – 6.3 µg mL^{-1}) with immobilized BSA-Lewis X (blue circles) or BSA (green squares), showing dose-dependent binding of Pl-TcdA1 to BSA-Lewis X and weak binding to BSA. The error bars represent standard deviations between three independent measurements, the individual data points are shown.

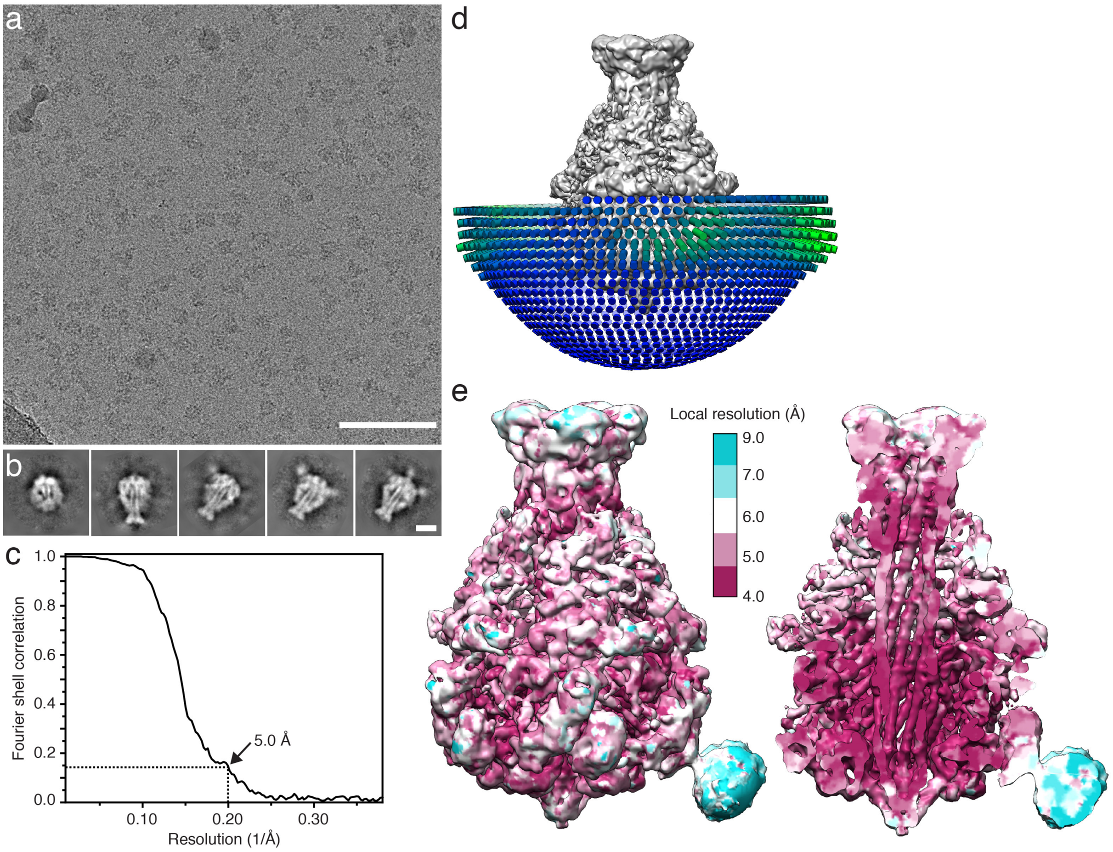

Supplementary Figure 3: Cryo-EM of Pl-TcdA1 and crosslinked BSA-Lewis X. a: Typical digital micrograph area of vitrified Pl-TcdA1-BSA-Lewis X complexes at a defocus of 2 µm and a total dose of 100 e^{-} $Å^{-2}$ acquired with a Falcon III direct electron detector. Scale bar, 100 nm. b: Representative reference-free 2D class averages obtained by ISAC and subsequently resampled to the original pixel size, refined and sharpened, using the Beautifier tool implemented in the SPHIRE software package. Scale bar, 10 nm. c: Fourier shell correlation (FSC) of the cryo-EM map (black curve). The 0.143 FSC cut-off criterion indicates that the cryo-EM map has an average resolution of 5.0 Å. d: Angular distribution for the final round of the refinement. Each stick represents a projection view. Size and color of the stick is proportional to the number of particles. e: Surface and cross-section of the cryo-EM density map colored according to the local resolution. The map sections corresponding to the Pl-TcdA1 pentamer and BSA-Lewis X are shown at different binarization thresholds.

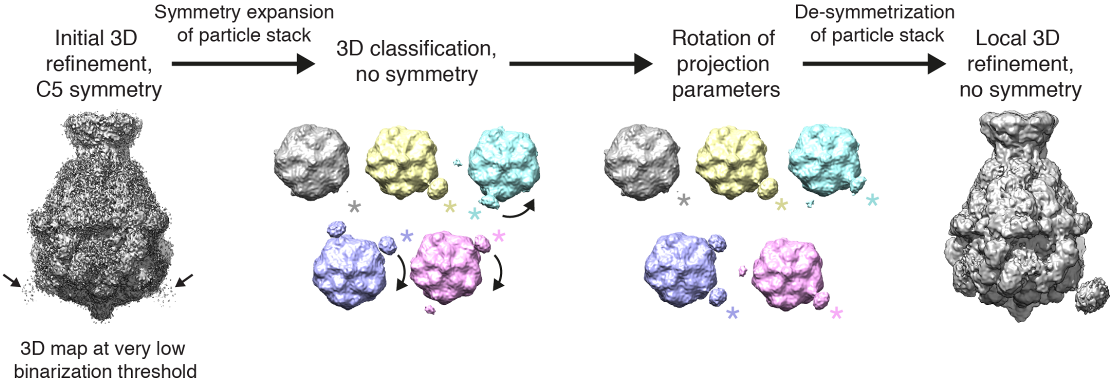

Supplementary Figure 4: Workflow of 3D classification and refinement for Pl-TcdA1-BSA-Lewis X. Initial 3D refinement with C5 symmetry resulted in weak density for BSA-Lewis X (arrows) which binds sub-stoichiometrically (left panel). Therefore, we performed symmetry expansion of the particle stack and 3D classification after 3D refinement. The 3D classes were rotated in 72º steps (indicated by curved arrows) so that the additional density corresponding to BSA-Lewis X was located in the same position and the projection parameters were adjusted accordingly. The stack was then de-symmetrized and a local 3D refinement without symmetry was performed, resulting in a map with BSA-Lewis X oriented at one interaction site of Pl-TcdA1 (right panel). The resolution of the final map is 5 Å.

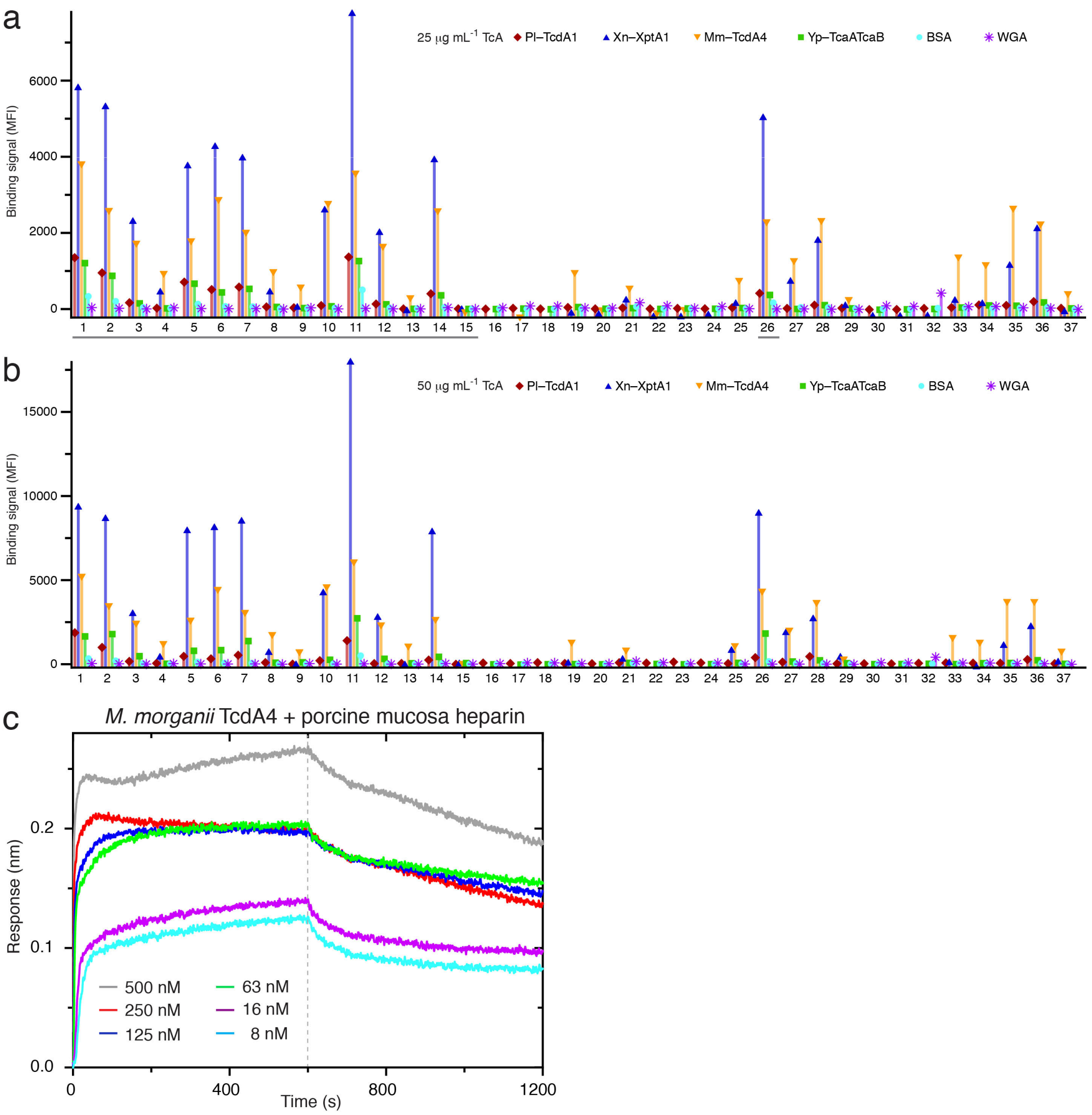

Supplementary Figure 5: Interaction of TcA with heparins. a,b: Glycan microarray showing the interaction of Pl-TcdA1 (red diamonds), Xn-XptA1 (blue triangles), Mm-TcdA4 (orange triangles), Yp-TcaA-TcaB (green squares) with various heparins and heparin-like glycans[1,2]. The protein concentration is 25 µg mL^{-1} (a) and 50 µg mL^{-1} (b), respectively. BSA (cyan circles) and WGA (purple stars) were used as controls. At positions 15 and 37, PBS buffer was spotted on the array as a negative control. The gray bars in (a) indicate the molecules that are presented in Figure 4a,b. c: BLI sensorgrams of Mm-TcdA4 (8 nM – 500 nM) with immobilized biotinylated porcine intestinal mucosa heparin. Association and dissociation phases are separated by a gray dashed line. The signal increase and decrease indicate association and dissociation of Mm-TcdA4 to the immobilized heparin.

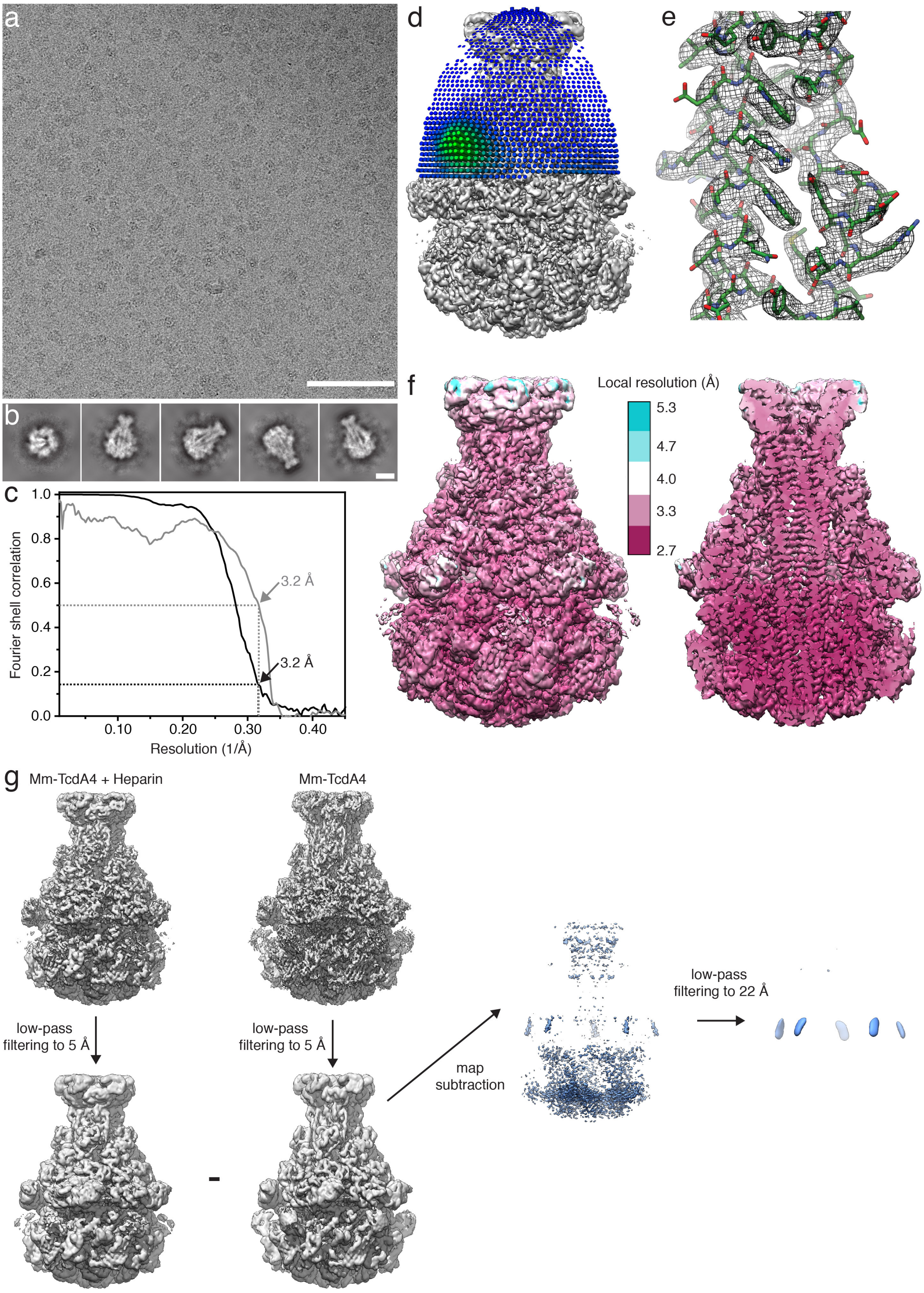

Supplementary Figure 6: Cryo-EM of Mm-TcdA4 in complex with heparin. a: Typical digital micrograph area of vitrified Mm-TcdA4-heparin complexes at a defocus of 2 μm and a total dose of 100 e^{-} Å^{-2} acquired with a Falcon III direct electron detector. Scale bar, 100 nm. b: Representative reference-free 2D class averages obtained by ISAC and subsequently resampled to the original pixel size, refined and sharpened,

using the Beautifier tool implemented in the SPHIRE software package. Scale bar, 10 nm. c: Fourier shell correlation (FSC) of the cryo-EM map (black curve). The 0.143 FSC cut-off criterion indicates that the cryo-EM map has an average resolution of 3.2 Å. The gray curve shows the FSC curve between the final map versus the atomic model. The 0.5 FSC cut-off criterion indicates a resolution of 3.2 Å. d: Angular distribution for the final round of the refinement. Each stick represents a projection view. Size and color of the stick is proportional to the number of particles. e: Superimposition of the cryo-EM density map and the model, shown for a representative area in the α-helical channel. f: Surface and cross-section of the cryo-EM density map colored according to the local resolution. g: Illustration of map subtraction to obtain the difference density map between Mm-TcdA4 in the absence of heparin[3] and Mm-TcdA4 in the presence of porcine intestinal mucosa heparin. After lowpass filtering of both maps to 5 Å, the map of Mm-TcdA4 is subtracted from the map of Mm-TcdA4 with heparin. The obtained difference density map is filtered to 22 Å resolution for illustrative purposes.

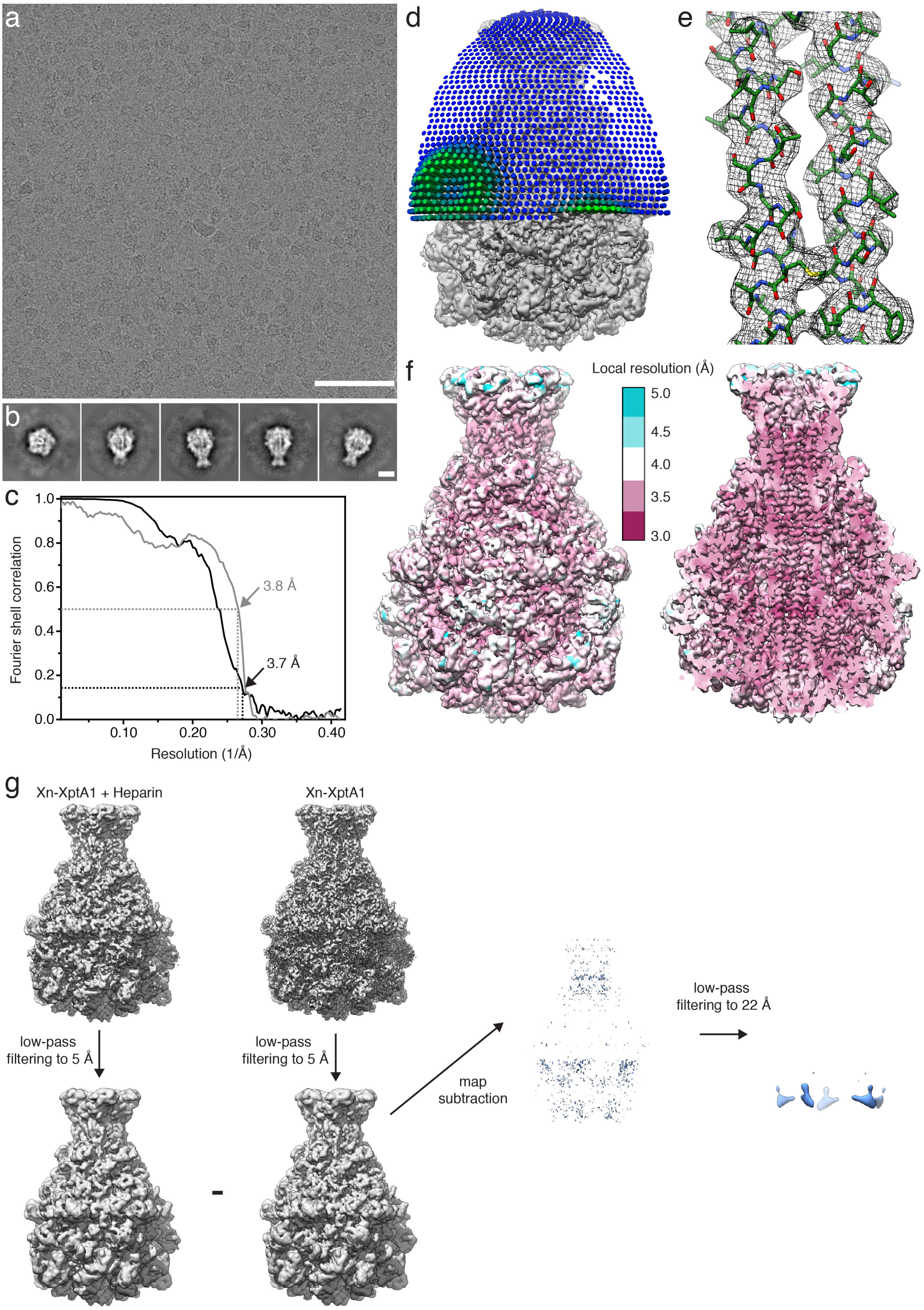

Supplementary Figure 7: Cryo-EM of Xn-XptA1 in complex with heparin. a: Typical digital micrograph area of vitrified Xn-XptA1-heparin complexes at a defocus of 2 μm and a total dose of 52 e^{-} $Å^{-2}$ acquired with a Falcon III direct electron detector. Scale bar, 100 nm. b: Representative reference-free 2D class averages obtained by ISAC and subsequently resampled to the original pixel size, refined and sharpened,

using the Beautifier tool implemented in the SPHIRE software package. Scale bar, 10 nm. c: Fourier shell correlation (FSC) of the cryo-EM map (black curve). The 0.143 FSC cut-off criterion indicates that the cryo-EM map has an average resolution of 3.7 Å. The gray curve shows the FSC curve between the final map versus the atomic model. The 0.5 FSC cut-off criterion indicates a resolution of 3.8 Å. d: Angular distribution for the final round of the refinement. Each stick represents a projection view. Size and color of the stick is proportional to the number of particles. e: Superimposition of the cryo-EM density map and the model, shown for a representative area in the α-helical channel. f: Surface and cross-section of the cryo-EM density map colored according to the local resolution. g: Illustration of map subtraction to obtain the difference density map between Xn-XptA1 in the absence of heparin[3] and Xn-XptA1 in the presence of porcine intestinal mucosa heparin. The workflow is analogous to Supplementary Figure 6g.

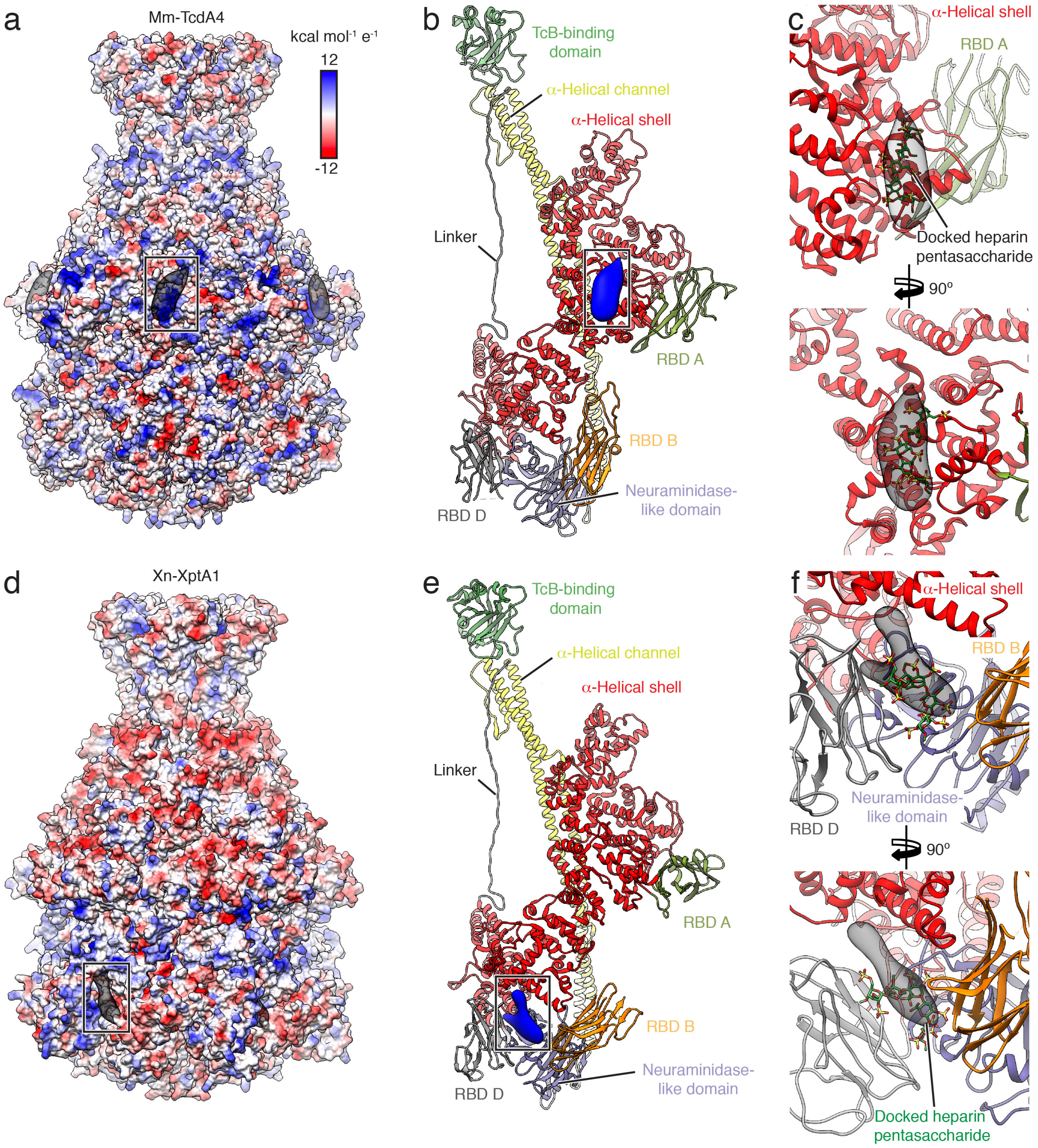

Supplementary Figure 8: Cryo-EM density of heparin mapped on the models of Mm-TcdA4 and Xn-XptA1, respectively. a,d: Surface representation of Mm-TcdA4 (a) and Xn-XptA1 (d) colored according to the Coulomb potential (kcal mol^{-1} e^{-1}) at pH 7.0. The boxes indicate the obtained difference density map (transparent gray) on one protomer. b,e: Model of one Mm-TcdA4 (b) and Xn-XptA1 (e) protomer with difference density map (blue). RBD: receptor-binding domain. c,f: Illustration of the docked heparin pentasaccharide on Mm-TcdA4 (c) and Xn-XptA1 (f). The docking solution that resulted in the best match with the difference density (transparent gray) is shown.

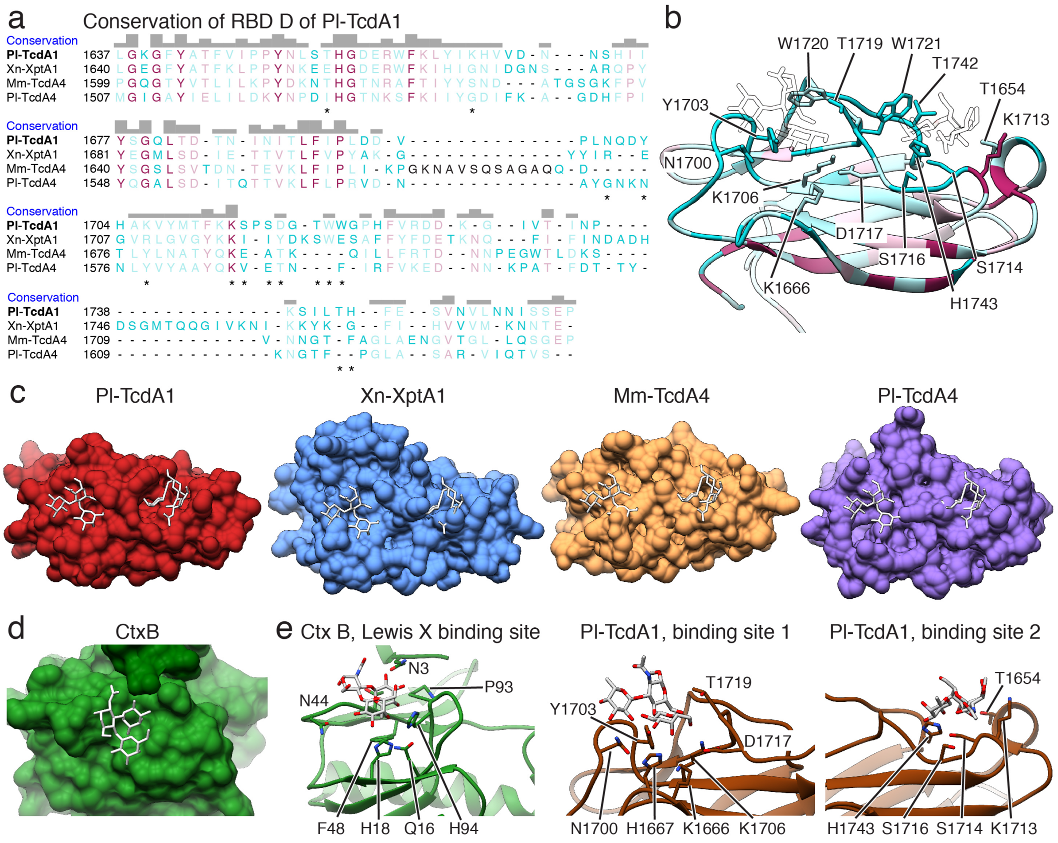

Supplementary Figure 9: No conservation of the Lewis X binding site of receptor binding domain D (RBD D) in different TcAs. a: Structure-based sequence alignment of RBD-D of Pl-TcdA1 and other TcAs[3]. Residues are colored according to their conservation ranging from minimum (cyan) to maximum (magenta). The black colored residues in Mm-TcdA4 are not resolved in the structure. The asterisks indicate residues that contact the docked Lewis X trisaccharides, as depicted in Fig. 3e and (b). b: RBD D of Pl-TcdA1 with mapped sequence conservation. The docked Lewis X trisaccharides are depicted in transparent. c: Comparison of the surface of RBD D of four different TcAs. The two Lewis X trisaccharides docked to Pl-TcdA1 are depicted as stick representations. Both binding pockets are only present in Pl-TcdA1. d: Surface representation of cholera toxin B subunit (CtxB) with bound Lewis X (PDB 6HJD). e: Comparison of the Lewis X binding site of CtxB and the two proposed Lewis X binding sites of Pl-TcdA1.

Supplementary Tables

Supplementary Table 1: Cryo-EM data collection, refinement and validation statistics for Mm-TcdA4-Heparin, Xn-XptA1-Heparin and Pl-TcdA1-BSA-Lewis X.

	Mm-TcdA4-Heparin (EMDB-10796) (PDB 6YEW)	Xn-XptA1-Heparin (EMDB-10797) (PDB 6YEY)	Pl-TcdA1-BSA-Lewis X (EMDB-10794)
**Data collection and processing**			
Magnification	59,000	120,000	59,000
Voltage (kV)	300	200	300
Electron exposure (e–/Å^2)	100	52	100
Defocus range (μm)	1.2 – 2.2	1.5 – 2.5	1.0 – 2.5
Pixel size (Å)	1.11	1.21	1.11
Symmetry imposed	C5	C5	C5/C1
Initial particle images (no.)	477,602	315,930	711,872
Final particle images (no.)	182,506	172,596	199,038
Map resolution (Å)	3.2	3.7	5.0
FSC threshold	0.143	0.143	0.143
Map resolution range (Å)	2.7 – 5.3	3.0 – 5.0	4.0 – 9.0
**Refinement**			
Initial model used (PDB code)	6RW9	6RW8	
Model resolution (Å)	3.2	3.8	
FSC threshold	0.5	0.5	
Map sharpening *B* factor (Å^2)	-127.06	-65.78	
Model composition			
Non-hydrogen atoms	88,700	93,500	
Protein residues	11,425	11,685	
Ligands			
B factors (Å^2)			
Protein	55.78	108.26	
Ligand			
R.m.s. deviations			
Bond lengths (Å)	0.011	0.006	
Bond angles (°)	0.901	0.810	
Validation			
MolProbity score	2.46	2.52	
Clashscore	13.89	6.63	
EMRinger score	2.22	1.80	
Poor rotamers (%)	2.83	8.11	
Ramachandran plot			
Favored (%)	92.42	92.62	
Allowed (%)	7.40	7.25	
Disallowed (%)	0.18	0.13	

Supplementary Table 2: Overview of all glycans on the microarray in Figure 2a. For additional information see Geissner *et al.*[4].

Glycan ID	Name	Structure	Compound no. Fig. 2a
5	Neu5Ac(a2-6)Gal(b1-4)GlcNAc(b1-3)Gal(b1-4)Glc(b1-1)aminohexanol	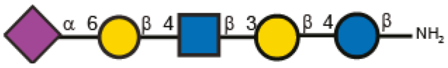	
6	Neu5Ac(a2-3)Gal(b1-3)GlcNAc(b1-3)Gal(b1-4)Glc(b1-1)aminohexanol	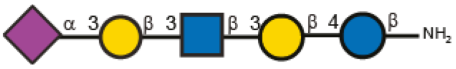	
7	Fuc(a1-3)[Neu5Ac(a2-3)Gal(b1-4)]GlcNAc(b1-3)Gal(b1-4)Glc(b1-1)aminohexanol	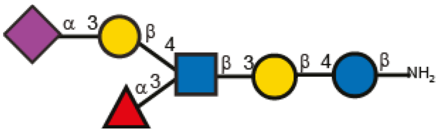	**4**
8	Neu5Ac(a2-6)Gal(b1-4)Glc(b1-1)aminohexanol	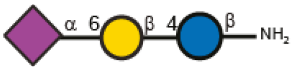	
9	Neu5Ac(a2-3)Gal(b1-4)Glc(b1-1)aminohexanol	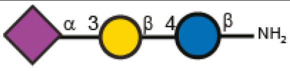	
10	Neu5Ac(a2-6)Gal(b1-4)GlcNAc-6-sulfate(b1-1)aminohexanol	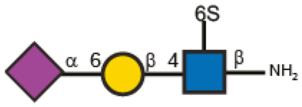	
11	Gal(b1-4)Glc(b1-1)aminohexanol	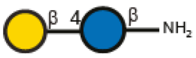	
12	Gal(b1-4)GlcNAc-6-sulfate(b1-1)aminohexanol	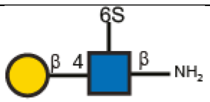	
69	D-Araf(a1-5)D-Araf(a1-1)aminopentanol	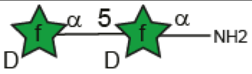	
70	D-Araf(a1-5)D-Araf(a1-3)[D-Araf(a1-5)D-Araf(a1-5)]D-Araf(a1-5)D-Araf(a1-1)aminopentanol	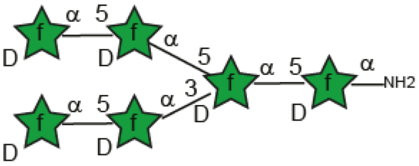	
71	D-Araf(a1-3)[D-Araf(a1-5)]D-Araf(a1-1)aminopentanol	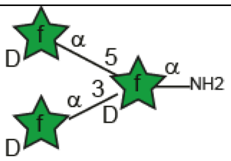	
72	D-Araf(a1-5)D-Araf(a1-5)D-Araf(a1-5)D-Araf(a1-	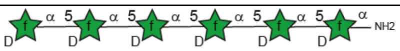	

	5)D-Araf(a1-5)D-Araf(a1-5)aminopentanol		
73	Col(a1-3)[Col(a1-6)]Glc(a1-4)Gal(a1-3)GlcNAc(b1-1)aminopentanol	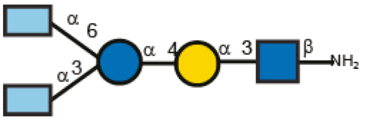	
74	ManNAc(b1-3)FucNAc(a1-3)GalNAc(a1-4)Gal(a1-1)aminopentanol	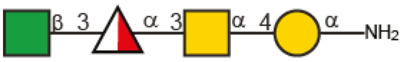	
75	GalNAc(a1-4)Gal(a1-1)aminopentanol	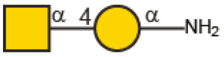	
76	GalNAc(b1-4)Gal(a1-1)aminopentanol	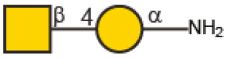	
77	FucNAc(a1-3)GalNAc(a1-4)Gal(a1-1)aminopentanol	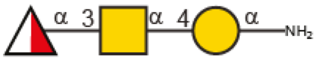	
78	FucNAc(b1-3)GalNAc(a1-4)Gal(a1-1)aminopentanol	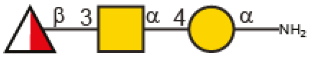	
80	GalNAc(b1-1)aminoethanol	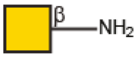	
81	FucNAc(a1-1)aminopentanol	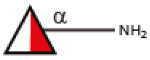	
82	Man(a1-2)Man(a1-2)[Gal(b1-4)]Man(a1-1)aminopentanol	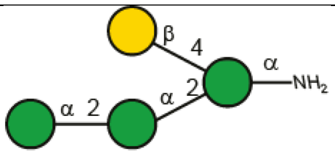	
83	Man(a1-2)Man(a1-2)[Gal(b1-4)]Man(a1-1)aminopentanol	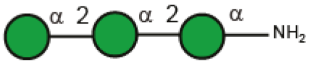	
84	Man(a1-2)Man(a1-2)Man(a1-1)aminopentanol	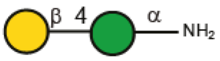	
85	Gal(b1-4)Man(a1-1)aminopentanol	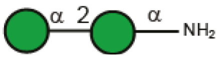	
90	Glc(b1-1)aminoethanol	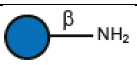	
91	GlcNAc(a1-2)Hep(a1-3)Hep(a1-5)Kdo(a2-1)aminopentanol	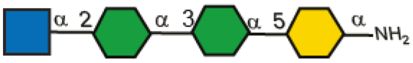	

92	Hep(a1-3)Hep(a1-5)Kdo(a2-1)aminopentanol	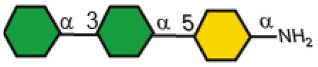	
93	Hep(a1-3)Hep(a1-5)[L-Ara4N(b1-8)]Kdo(a2-1)aminopentanol	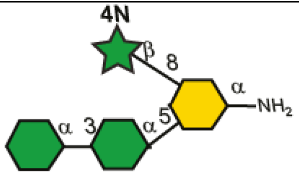	
94	Hep(a1-7)Hep(a1-3)Hep(a1-5)Kdo(a2-1)aminopentanol	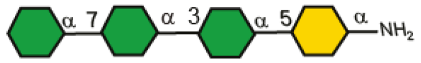	
95	Hep(a1-2)Hep(a1-3)Hep(a1-5)Kdo(a2-1)aminopentanol	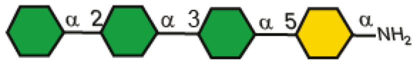	
96	Hep(a1-5)Kdo(a2-1)aminopentanol	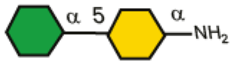	
97	Hep(a1-7)Hep(a1-3)Hep(a1-1)aminopentanol	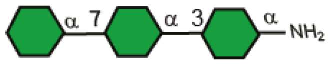	
98	Kdo(a2-8)Kdo(a2-4)Kdo(a2-1)aminopentanol	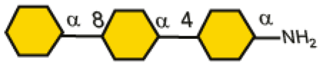	
99	Kdo(a2-1)aminopentanol	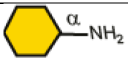	
100	Hep(a1-1)aminopentanol	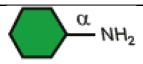	
101	Glc(b1-1)aminopentanol	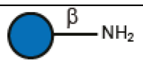	
102	D-FucNAc(b1-1)aminopentanol	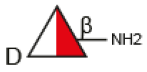	
103	D-FucNAc(b1-1)aminopentanol	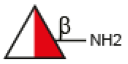	
104	FucNAc(b1-1)aminopentanol	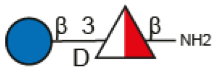	
105	FucNAc(b1-1)aminopentanol	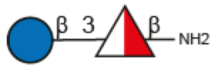	
153	Gal(b1-3)GalNAc(a1-1)aminopentanol	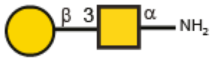	
154	Fuc(a1-3)[Gal(b1-4)]GlcNAc(b1-1)aminopentanol	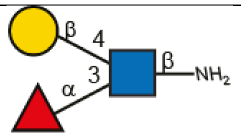	**11**

155	Neu5Ac(a2-6)GalNAc(a1-1)aminopentanol	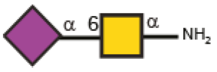	
156	Gal(b1-4)[Gal(b1-4)Glc(b1-6)]GlcNAc(b1-1)aminopentanol	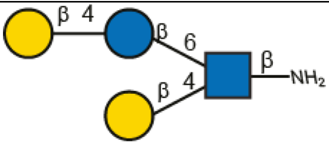	
157	Fuc(a1-3)[Fuc(a1-2)Gal(b1-4)]GlcNAc(b1-1)aminopentanol	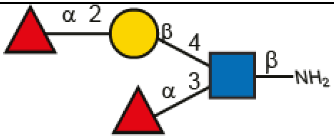	**5**
158	Gal(b1-3)[Fuc(a1-4)]GlcNAc(b1-1)aminopentanol	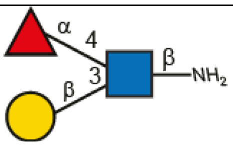	**12**
159	Fuc(a1-2)Gal(b1-3)[Fuc(a1-4)]GlcNAc(b1-1)aminopentanol	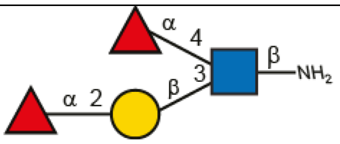	**6**
160	Gal-2,3-Pyruvate(a1-1)aminopentanol	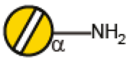	
161	Gal(a1-3)Gal(b1-4)Glc(b1-1)aminopentanol	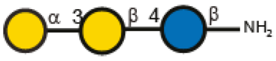	
162	Gal(a1-3)Gal(b1-4)GlcNAc(b1-1)aminopentanol	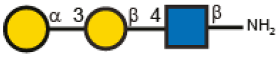	
163	Gal(a1-3)Gal(b1-4)GlcNAc(b1-3)Gal(b1-4)Glc(b1-1)aminopentanol	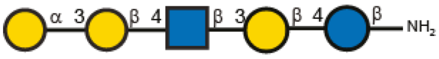	
164	Gal(b1-4)GlcNAc(b1-3)Gal(b1-4)Glc(b1-1)aminopentanol	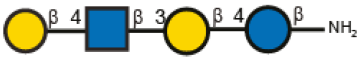	
165	Fuc(a1-2)Gal(b1-3)GlcNAc(b1-3)Gal(b1-4)Glc(b1-1)aminopentanol	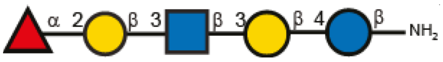	
166	Gal(b1-3)GlcNAc(b1-3)Gal(b1-4)Glc(b1-1)aminopentanol	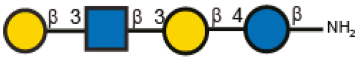	
167	Rha(a1-1)aminopentanol	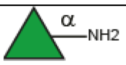	
168	Rha(a1-3)Glc(b1-1)aminopentanol	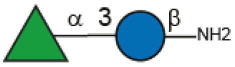	

169	Glc(a1-2)Glc(a1-1)aminopentanol	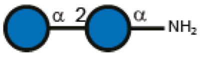	
170	Glc(b1-4)Glc(a1-2)Glc(a1-1)aminopentanol	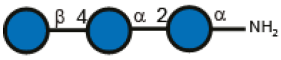	
171	Rha(a1-3)Glc(b1-4)Glc(a1-1)aminopentanol	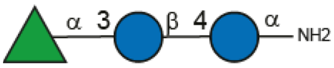	
172	Gal(b1-3)GalNAc(b1-3)Gal(a1-4)Gal(b1-4)Glc(b1-1)aminopentanol	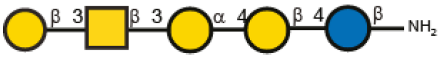	
173	Neu5Ac(a2-8)Neu5Ac(a2-3)[GalNAc(b1-4)]Gal(b1-4)Glc(b1-1)aminopentanol	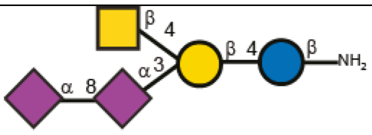	
174	Gal(a1-4)Gal(b1-4)Glc(b1-1)aminopentanol	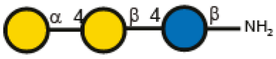	
175	GalNAc(a1-1)AminoLinker2	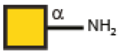	
176	Fuc(a1-3)[Gal(b1-4)]GlcNAc(b1-1)AminoLinker2	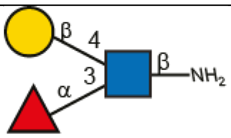	7
177	GlcNAc(a1-2)Hep(a1-3)Hep(a1-1)aminopentanol	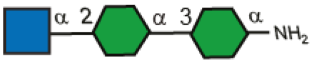	
178	Hep(a1-3)Hep(a1-1)aminopentanol	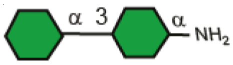	
179	Gal(b1-4)Glc(b1-1)aminopentanol	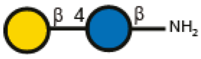	
180	GalNAc(b1-4)Gal(b1-4)Glc(b1-1)aminopentanol	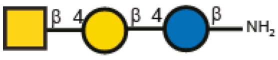	
181	Neu5Ac(a2-3)Gal(b1-4)Glc(b1-1)aminopentanol	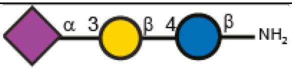	
182	GalNAc-4-sulfate(b1-1)aminopentanol	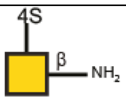	
183	IdoA-2,4-disulfate(a1-1)aminopentanol	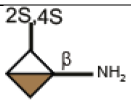	
184	IdoA(a1-3)GalNAc-4-sulfate(b1-1)aminopentanol	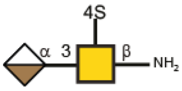	

185	IdoA-2-sulfate(a1-3)GalNAc-4-sulfate(b1-1)aminopentanol	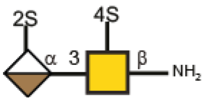	
186	IdoA(a1-3)GalNAc(b1-1)aminopentanol	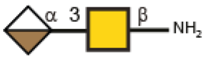	
187	GlcA(b1-4)Glc(b1-3)GlcA(b1-4)Glc(b1-1)aminoethanol	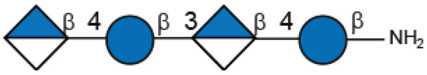	
188	Glc(b1-3)GlcA(b1-4)Glc(b1-1)aminoethanol	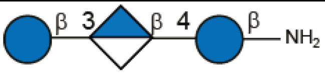	
189	GalNAc(a1-1)Thr-Linker	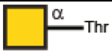	
190	Glc(b1-3)Glc(b1-3)[Glc(b1-6)]Glc(b1-3)Glc(b1-1)aminopentanol	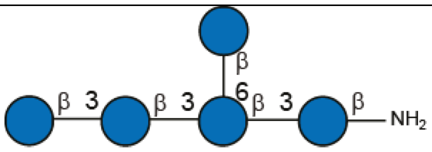	
191	Glc(b1-3)Glc(b1-3)[Glc(b1-6)]Glc(b1-3)Glc(b1-3)Glc(b1-3)Glc(b1-3)Glc(b1-3)Glc(b1-1)aminopentanol	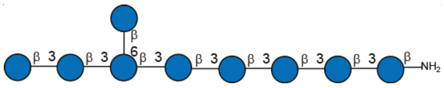	
192	Glc(b1-3)Glc(b1-3)[Glc(b1-6)]Glc(b1-3)Glc(b1-3)Glc(b1-3)Glc(b1-3)Glc(b1-3)Glc(b1-3)Glc(b1-3)Glc(b1-3)Glc(b1-3)Glc(b1-1)aminopentanol	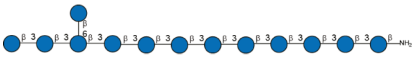	
193	Glc(b1-3)Glc(b1-3)Glc(b1-3)Glc(b1-3)Glc(b1-3)Glc(b1-3)Glc(b1-3)Glc(b1-3)Glc(b1-3)Glc(b1-3)Glc(b1-3)Glc(b1-1)aminopentanol	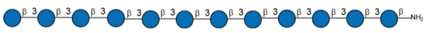	
194	L-PneNAc(a1-2)GlcA(b1-3)FucNAc(a1-3)D-FucNAc(b1-1)aminopentanol	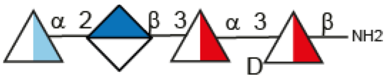	
195	Mixture of: D-6d-xylHexpNAc-4-ulo(b1-1)aminopentanol and D-	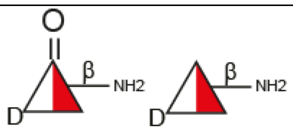	

	FucNAc(b1-1)aminopentanol		
196	Mixture of: FucNAc(a1-3)D-6d-xylHexpNAc-4-ulo(b1-1)aminopentanol and FucNAc(a1-3)D-FucNAc(b1-1)aminopentanol	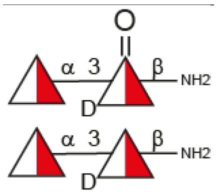	
197	FucNAc(a1-3)D-FucNAc(b1-1)aminopentanol	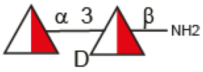	
198	GlcA(b1-4)FucNAc(a1-1)aminopentanol	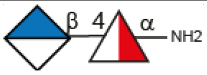	
199	Glc(b1-3)FucNAc(a1-1)aminopentanol	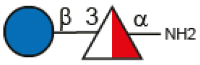	
200	L-PneNAc(a1-2)GlcA(b1-1)aminopentanol	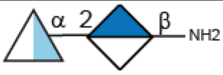	
201	L-PneNAc(a1-1)aminopentanol	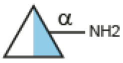	
202	L-PneNAc(b1-1)aminopentanol	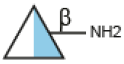	
203	Gal(b1-4)[Glc(b1-6)]GlcNAc(b1-3)Gal(b1-1)aminopentanol	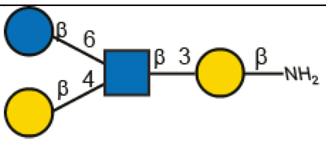	
204	Glc(a1-4)Gal(a1-4)GlcA(b1-4)Glc(b1-1)aminoethanol	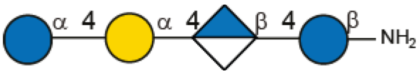	
205	Glc(a1-4)Gal(a1-1)aminoethanol	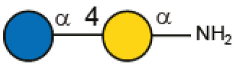	
206	GlcA(b1-4)Glc(b1-4)Glc(a1-4)Gal(a1-1)aminoethanol	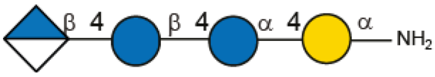	
207	Glc(a1-4)Gal(a1-4)GlcA(b1-4)Glc(b1-1)aminopentanol	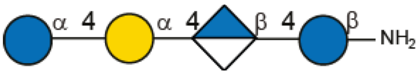	
208	Gal(a1-4)GlcA(b1-4)Glc(b1-4)Glc(a1-1)aminopentanol	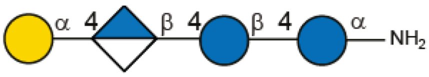	

209	GlcA(b1-4)Glc(b1-4)Glc(a1-4)Gal(a1-1)aminopentanol	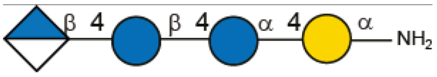	
210	Glc(b1-4)Glc(a1-4)Gal(a1-4)GlcA(b1-1)aminopentanol	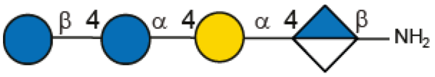	
211	Xyl(b1-4)Xyl(b1-1)aminopentanol	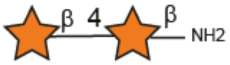	
212	Xyl(b1-4)Xyl(b1-4)Xyl(b1-4)Xyl(b1-1)aminopentanol	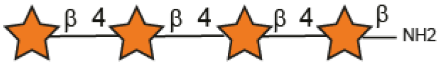	
213	Xyl(b1-4)Xyl(b1-4)Xyl(b1-4)Xyl(b1-4)Xyl(b1-4)Xyl(b1-1)aminopentanol	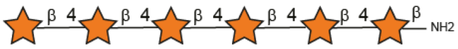	
214	Xyl(b1-4)Xyl(b1-4)Xyl(b1-4)Xyl(b1-4)Xyl(b1-4)Xyl(b1-4)Xyl(b1-4)Xyl(b1-1)aminopentanol	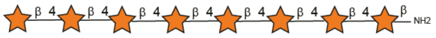	
215	Glc(b1-4)Glc(b1-4)Glc(b1-4)Glc(b1-1)aminopentanol	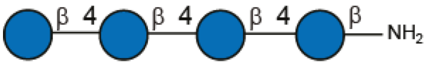	
216	Glc(b1-3)GlcA(b1-4)Glc(b1-1)aminopentanol	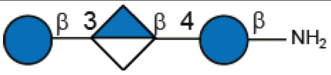	
217	GlcA(b1-4)Glc(b1-1)aminoethanol	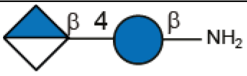	
218	Glc(b1-3)GlcA(b1-1)aminoethanol	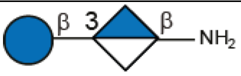	
219	ManNAc(b1-3)FucNAc(a1-3)GalNAc(a1-4)Gal-2,3-pyruvate(a1-1)aminopentanol	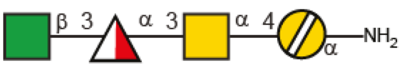	
220	GlcA(b1-1)aminoethanol	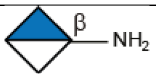	
225	Glc(a1-4)GalNAc(b1-4)Man(a1-1)aminopentanol	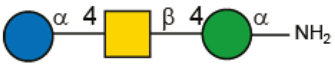	

226	Glc(a1-4)GalNAc(b1-4)[Man(a1-2)Man(a1-6)]Man(a1-1)aminopentanol	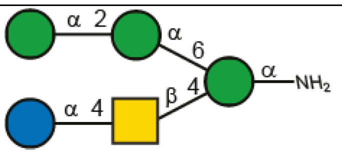	
227	Glc(a1-4)GalNAc(b1-4)[Man-6-PEtN(a1-2)Man(a1-6)]Man(a1-1)aminopentanol	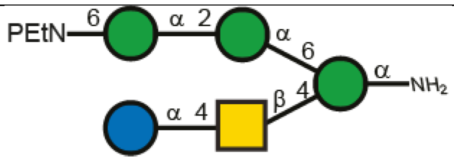	
229	GalNAc(b1-4)Man(a1-1)aminopentanol	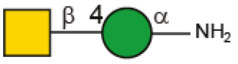	
230	GalNAc(b1-4)[Man-6-PEtN(a1-2)Man(a1-6)]Man(a1-1)aminopentanol	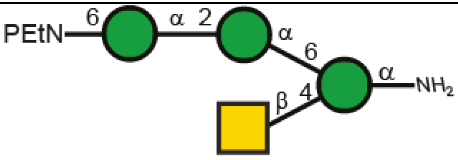	
231	GalNAc(b1-4)[Man(a1-2)Man(a1-6)]Man(a1-1)aminopentanol	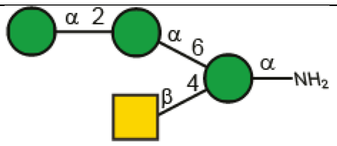	
232	GalNAc(b1-4)[Man-6-PEtN(a1-2)Man(a1-6)]Man-2-PEtN(a1-1)aminopentanol	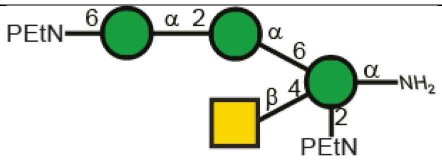	
233	GalNAc(b1-4)Man(a1-1)aminododecanol	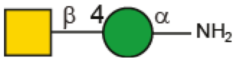	
234	GalNAc(b1-4)Man(a1-1)p-aminocyclohexanol	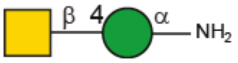	
235	GlcA(b1-4)Glc(b1-3)GlcA(b1-1)aminoethanol	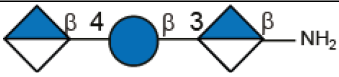	
236	GlcA(b1-4)Glc(b1-3)Glc(b1-4)Glc(b1-1)aminoethano and/or Glc(b1-3)Glc(b1-3)GlcA(b1-4)Glc(b1-1)aminoethanol	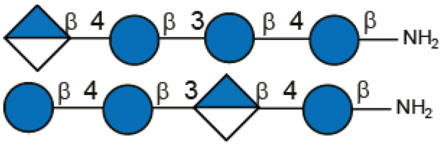	
237	Man(a1-1)aminopentanol	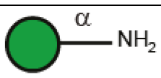	
238	GlcNAc-6-P-phosphoaminopentanol(a1-3)GlcNAc-6-P-phosphoaminopentanol(a1-2)glyceric acid	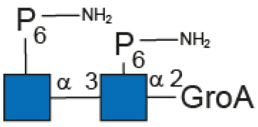	

239	GlcA(a1-3)Gal(a1-3)ManNAc(b1-4)Glc(b1-4)Glc(a1-1)aminopentanol	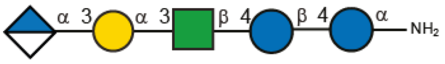	
240	GlcA(a1-3)Gal(a1-3)ManNAc-6-acetate(b1-4)Glc(b1-4)Glc(a1-1)aminopentanol	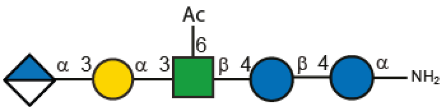	
241	GlcA(a1-3)Gal(a1-1)aminopentanol	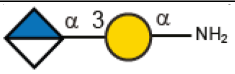	
242	Glc(b1-4)Glc(a1-1)aminopentanol	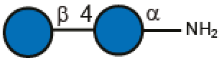	
243	ManNAc(b1-4)Glc(b1-4)Glc(a1-1)aminopentanol	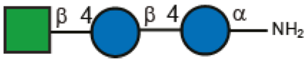	
244	GalNAc(b1-3)GalNAc(b1-1)aminopentanol	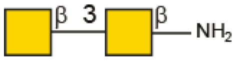	
245	Glc(b1-4)Gal(b1-4)Glc(b1-1)aminopentanol	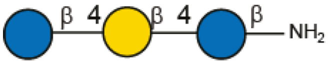	
247	Rha(a1-3)[Rha(a1-3)Glc(b1-4)]Glc(a1-2)Glc(a1-1)aminopentanol	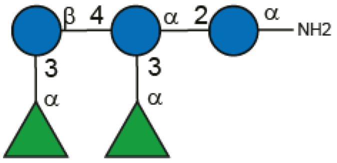	
248	GlcNAc(a1-3)GlcNAc-6-P-phosphoaminopentanol(a1-2)glyceric acid	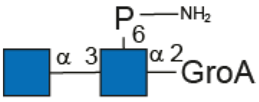	
249	GlcNAc(a1-3)GlcNAc[(a1-2)glyceric acid](6-P-6)GlcNAc(a1-3)GlcNAc-6-P-phosphoaminopentanol(a1-2)glyceric acid	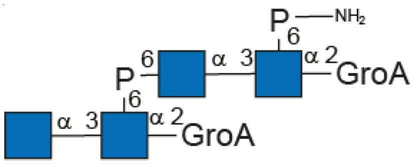	
250	Man(a1-2)Man(a1-2)[Gal(b1-4)]Man(a1-1)aminoethanol	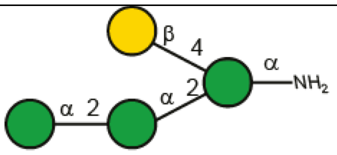	
251	Glc(b1-3)Gal(b1-4)Man(a1-1)aminopentanol	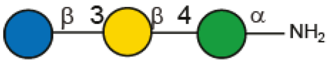	
252	Rha(a1-2)Rha(a1-2)Rha(a1-1)aminopentanol	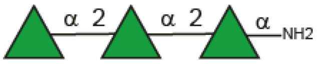	

253	GalNAc-2,3-Oxazolidinone(a1-4)GalNAc-2,3-Oxazolidinone(a1-1)aminopentanol	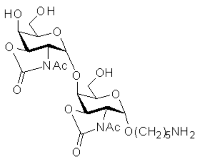	
254	Glc(a1-2)Glc(a1-3)[FucNAc(a1-3)GalNAc(b1-4)]ManNAcA(b1-1)aminopentanol	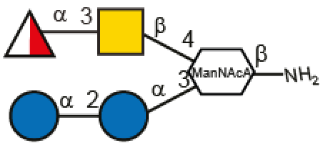	
255	Glc(a1-2)Glc(a1-3)[Gal(a1-3)FucNAc(a1-3)GalNAc(b1-4)]ManNAcA(b1-1)aminopentanol	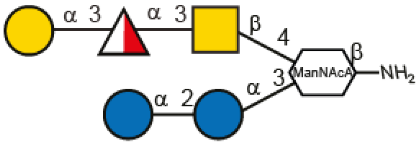	
256	D-Araf(a1-3)[D-Araf(a1-5)]D-Araf(a1-5)D-Araf(a1-1)aminopentanol	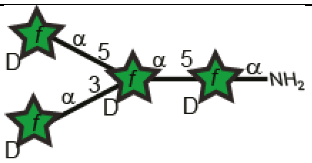	
257	Man(a1-5)D-Araf(a1-3)[Man(a1-5)D-Araf(a1-5)]D-Araf(a1-5)D-Araf(a1-1)aminopentanol	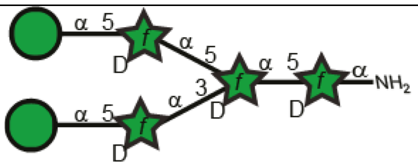	
258	Man(a1-5)D-Araf(a1-3)[Man(a1-5)D-Araf(a1-5)]D-Araf(a1-5)D-Araf(a1-1)aminopentanol	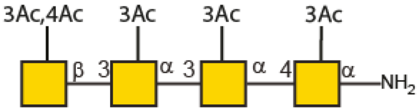	

Supplementary Table 3: Overview of all glycans on the microarrays in Supplementary Figure 5a,b. For additional information see Geissner *et al.*[4].

**Glycan ID**	**Name**	**Structure**	**Compound no. Fig. S5a/b**
5	Neu5Ac(a2-6)Gal(b1-4)GlcNAc(b1-3)Gal(b1-4)Glc(b1-1)aminohexanol	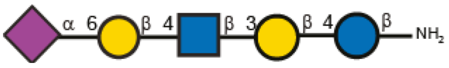	**16**
6	Neu5Ac(a2-3)Gal(b1-3)GlcNAc(b1-3)Gal(b1-4)Glc(b1-1)aminohexanol	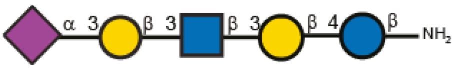	**17**
7	Fuc(a1-3)[Neu5Ac(a2-3)Gal(b1-4)]GlcNAc(b1-3)Gal(b1-4)Glc(b1-1)aminohexanol	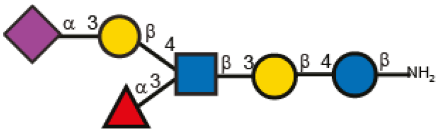	**18**
8	Neu5Ac(a2-6)Gal(b1-4)Glc(b1-1)aminohexanol	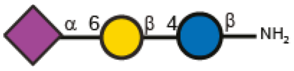	**19**
9	Neu5Ac(a2-3)Gal(b1-4)Glc(b1-1)aminohexanol	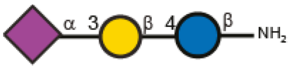	**20**
10	Neu5Ac(a2-6)Gal(b1-4)GlcNAc-6-sulfate(b1-1)aminohexanol	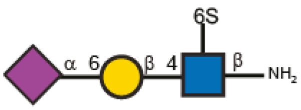	**21**
155	Neu5Ac(a2-6)GalNAc(a1-1)aminopentanol	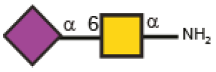	**22**
173	Neu5Ac(a2-8)Neu5Ac(a2-3)[GalNAc(b1-4)]Gal(b1-4)Glc(b1-1)aminopentanol	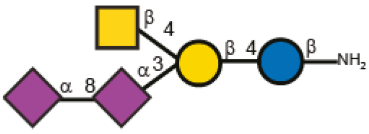	**23**
181	Neu5Ac(a2-3)Gal(b1-4)Glc(b1-1)aminopentanol	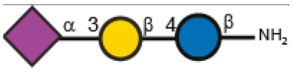	**24**

182	GalNAc-4-sulfate(b1-1)aminopentanol	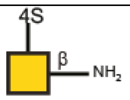	**25**
183	IdoA-2,4-disulfate(a1-1)aminopentanol	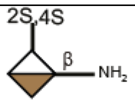	**26**
184	IdoA(a1-3)GalNAc-4-sulfate(b1-1)aminopentanol	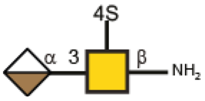	**27**
185	IdoA-2-sulfate(a1-3)GalNAc-4-sulfate(b1-1)aminopentanol	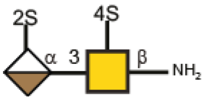	**28**
186	IdoA(a1-3)GalNAc(b1-1)aminopentanol	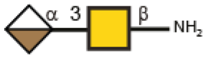	**29**
269	Gal(b1-4)GlcNAc(b1-3)Gal(b1-4)GlcNAc(b1-1)aminopentanol	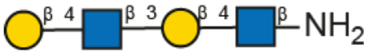	**30**
270	Gal(b1-4)GlcNAc(b1-3)[Gal(b1-4)GlcNAc(b1-6)]Gal(b1-4)GlcNAc(b1-1)aminopentanol	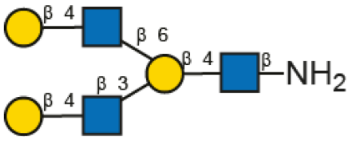	**31**
271	Gal(b1-4)GlcNAc(b1-3)Gal(b1-4)GlcNAc(b1-3)Gal(b1-4)GlcNAc(b1-1)aminopentanol	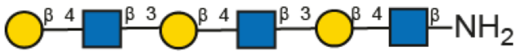	**32**
272	Gal-6-sulfate(b1-4)GlcNAc(b1-3)Gal-6-sulfate(b1-4)GlcNAc(b1-1)aminopentanol	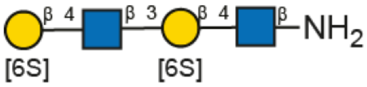	**33**
273	Gal(b1-4)GlcNAc-6-sulfate(b1-3)Gal(b1-4)GlcNAc-6-sulfate(b1-1)aminopentanol	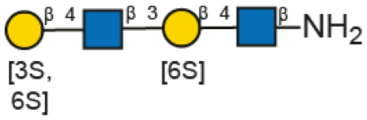	**34**

274	Gal-3,6-disulfate(b1-4)GlcNAc(b1-3)Gal-6-sulfate(b1-4)GlcNAc(b1-1)aminopentanol	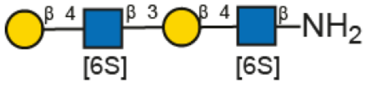	**35**
275	Gal-6-sulfate(b1-4)GlcNAc-6-sulfate(b1-3)Gal-6-sulfate(b1-4)GlcNAc-6-sulfate(b1-1)aminopentanol	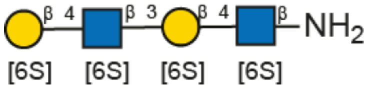	**36**

Supplementary References

1. de Paz, J. L., Noti, C. & Seeberger, P. H. Microarrays of synthetic heparin oligosaccharides. *J. Am. Chem. Soc.* **128,** 2766–2767 (2006).
2. Noti, C., de Paz, J. L., Polito, L. & Seeberger, P. H. Preparation and use of microarrays containing synthetic heparin oligosaccharides for the rapid analysis of heparin-protein interactions. *Chemistry* **12,** 8664–8686 (2006).
3. Leidreiter, F. *et al.* Common architecture of Tc toxins from human and insect pathogenic bacteria. *Sci Adv* **5,** eaax6497 (2019).
4. Geissner, A. *et al.* Microbe-focused glycan array screening platform. *Proc. Natl. Acad. Sci. U.S.A.* **116,** 1958–1967 (2019).
