# Supplementary material for: Glycan-dependent cell adhesion mechanism of Tc toxins
Source: Nat Commun. 2020 Jun 1;11:2694. doi: 10.1038/s41467-020-16536-7 (PMC7264150; doi:10.1038/s41467-020-16536-7)
Supplement: Supplementary file 3 — Description of Additional Supplementary Information [file 41467_2020_16536_MOESM3_ESM.pdf]

Description of Additional Supplementary Files

File Name: Supplementary Movie 1
Description: Cryo-EM density map of Pl-TcdA1/BSA-Lewis X. The map section corresponding to BSA-Lewis X is displayed at a lower binarization threshold. A surface representation of RBD D and the docked BSA-Lewis X molecules are shown.

File Name: Supplementary Movie 2
Description: Cryo-EM density map of Mm-TcdA4/heparin with the fitted atomic model.

File Name: Supplementary Movie 3
Description: Cryo-EM density map of Xn-XptA1/heparin with the fitted atomic model.
