# Supplementary material for: Glycan-dependent cell adhesion mechanism of Tc toxins
Source: Nat Commun. 2020 Jun 1;11:2694. doi: 10.1038/s41467-020-16536-7 (PMC7264150; doi:10.1038/s41467-020-16536-7)
Supplement: Supplementary file 7 — Reporting summary [file 41467_2020_16536_MOESM7_ESM.pdf]

nature research

Corresponding author(s): Stefan Raunser

Last updated by author(s): May 3, 2020

Reporting Summary

Nature Research wishes to improve the reproducibility of the work that we publish. This form provides structure for consistency and transparency in reporting. For further information on Nature Research policies, see Authors & Referees and the Editorial Policy Checklist .

Statistics

For all statistical analyses, confirm that the following items are present in the figure legend, table legend, main text, or Methods section.

n/a	Confirmed	
☐	☒	The exact sample size (n) for each experimental group/condition, given as a discrete number and unit of measurement
☐	☒	A statement on whether measurements were taken from distinct samples or whether the same sample was measured repeatedly
☐	☒	The statistical test(s) used AND whether they are one- or two-sided<br>*Only common tests should be described solely by name; describe more complex techniques in the Methods section.*
☒	☐	A description of all covariates tested
☒	☐	A description of any assumptions or corrections, such as tests of normality and adjustment for multiple comparisons
☐	☒	A full description of the statistical parameters including central tendency (e.g. means) or other basic estimates (e.g. regression coefficient) AND variation (e.g. standard deviation) or associated estimates of uncertainty (e.g. confidence intervals)
☒	☐	For null hypothesis testing, the test statistic (e.g. F, t, r) with confidence intervals, effect sizes, degrees of freedom and P value noted<br>*Give P values as exact values whenever suitable.*
☒	☐	For Bayesian analysis, information on the choice of priors and Markov chain Monte Carlo settings
☒	☐	For hierarchical and complex designs, identification of the appropriate level for tests and full reporting of outcomes
☒	☐	Estimates of effect sizes (e.g. Cohen's d, Pearson's r), indicating how they were calculated

Our web collection on statistics for biologists contains articles on many of the points above.

Software and code

Policy information about availability of computer code

Data collection	Cryo-EM data were collected with EPU version 1.8 and 1.9 (Thermo Fisher Scientific). Fluorescence microscopy images were collected with ZEN Blue.
Data analysis	Cryo-EM data were processed with SPHIRE, version 1.0 and 1.3. Fluorescence images were analyzed with ImageJ. Glycan array data were analyzed with the FlexAnalysis software (Bruker).

For manuscripts utilizing custom algorithms or software that are central to the research but not yet described in published literature, software must be made available to editors/reviewers. We strongly encourage code deposition in a community repository (e.g. GitHub). See the Nature Research guidelines for submitting code & software for further information.

Data

Policy information about availability of data

All manuscripts must include a data availability statement. This statement should provide the following information, where applicable:

- Accession codes, unique identifiers, or web links for publicly available datasets
- A list of figures that have associated raw data
- A description of any restrictions on data availability

The coordinates for the EM structures of Pl-TcdA1-BSA-Lewis X, Mm-TcdA4-heparin and Xn-XptA1-heparin have been deposited in the Electron Microscopy Data Bank under accession numbers 10794, 10796 and 10797, respectively. The models of Mm-TcdA4 refined against the EM density map of Mm-TcdA4-heparin and Xn-XptA1 refined against the EM density map of Xn-XptA1-heparin have been deposited in the Protein Data Bank under accession numbers 6YEW and 6YEY, respectively. The source data underlying Figs 1b,c, 2a,b, 4a and Supplementary Figs 2d and 5a,b are provided as a Source Data file. Other data are available from the corresponding author upon reasonable request.

Field-specific reporting

Please select the one below that is the best fit for your research. If you are not sure, read the appropriate sections before making your selection.

- [x] Life sciences
- [ ] Behavioural & social sciences
- [ ] Ecological, evolutionary & environmental sciences

For a reference copy of the document with all sections, see nature.com/documents/nr-reporting-summary-flat.pdf

Life sciences study design

All studies must disclose on these points even when the disclosure is negative.

Sample size	Flow cytometry (Fig. 1c): 50000 events were counted per sample.<br>BLI experiments: 7 samples (Fig. 2d) or 6 samples (Supplementary Fig. 5c) were chosen per measurement, determined by the design of the Octet Red 384 instrument enabling 8 parallel measurements with one data point as the negative control. The global fit included all curves after double reference correction. No data were excluded, in Supplementary Fig. 5c one spectrometer of the Octet was not used.
Data exclusions	Fluorescence microscopy: No data were excluded from the analysis.<br>BLI experiments: No data were excluded from the analysis.
Replication	Fig. 1b: Three replicates were applied for every data set.<br>Fig. 2a,b: Four replicates were applied.<br>Supplementary Figure 2d: Three replicated were applied.
Randomization	Samples were not randomized.
Blinding	The investigators were not blinded because every experiment was performed by one person.

Reporting for specific materials, systems and methods

We require information from authors about some types of materials, experimental systems and methods used in many studies. Here, indicate whether each material, system or method listed is relevant to your study. If you are not sure if a list item applies to your research, read the appropriate section before selecting a response.

Materials & experimental systems

n/a	Involved in the study	
x		Antibodies
	x	Eukaryotic cell lines
x		Palaeontology
x		Animals and other organisms
x		Human research participants
x		Clinical data

Methods

n/a	Involved in the study	
x		ChIP-seq
	x	Flow cytometry
x		MRI-based neuroimaging

Eukaryotic cell lines

Policy information about cell lines

Cell line source(s)	HEK 293T cells and HEK 293GnTi- cells were obtained from ThermoFisher.
Authentication	Cell lines were not authenticated.
Mycoplasma contamination	Cells were not tested for Mycoplasma contamination.
Commonly misidentified lines (See ICLAC register)	No commonly misidentified cell lines were used.

Flow Cytometry

Plots

Confirm that:

- [x] The axis labels state the marker and fluorochrome used (e.g. CD4-FITC).
- [x] The axis scales are clearly visible. Include numbers along axes only for bottom left plot of group (a 'group' is an analysis of identical markers).
- [ ] All plots are contour plots with outliers or pseudocolor plots.
- [x] A numerical value for number of cells or percentage (with statistics) is provided.

Methodology

Sample preparation	HEK 293T cells were deglycosylated, incubated with Alexa488-labeled toxin (Pl-TcdA1) and washed before analysis, as stated in the methods section. Cells were not fixed before flow cytometry.
Instrument	BD LSR II
Software	Data collection: FACS Diva<br>Data analysis: FlowJo version 10.1
Cell population abundance	n.a., we did not fractionate and sort the cells.
Gating strategy	Initially, cells were separated from debries by FCS/SSC gating. Subsequently, the entire cell population in the samples without Pl-TcdA1 was identified as non-fluorescent cells and the gating window was set accordingly.<br>The gating strategy is shown in the Source Data file.

- [x] Tick this box to confirm that a figure exemplifying the gating strategy is provided in the Supplementary Information.
